# Comparison study of microarray meta-analysis methods

**DOI:** 10.1186/1471-2105-11-408

**Published:** 2010-08-03

**Authors:** Anna Campain, Yee Hwa Yang

**Affiliations:** 1 School of Mathematics and Statistics, Center of Mathematical Biology, University of Sydney, F07 Sydney, NSW 2006, Australia

## Abstract

**Background:**

Meta-analysis methods exist for combining multiple microarray datasets. However, there are a wide range of issues associated with microarray meta-analysis and a limited ability to compare the performance of different meta-analysis methods.

**Results:**

We compare eight meta-analysis methods, five existing methods, two naive methods and a novel approach (mDEDS). Comparisons are performed using simulated data and two biological case studies with varying degrees of meta-analysis complexity. The performance of meta-analysis methods is assessed via ROC curves and prediction accuracy where applicable.

**Conclusions:**

Existing meta-analysis methods vary in their ability to perform successful meta-analysis. This success is very dependent on the complexity of the data and type of analysis. Our proposed method, mDEDS, performs competitively as a meta-analysis tool even as complexity increases. Because of the varying abilities of compared meta-analysis methods, care should be taken when considering the meta-analysis method used for particular research.

## Background

Many researchers have embraced microarray technology. Due to extensive usage of microarray technology, in recent years there has been an explosion in publicly available datasets. Examples of such repositories include Gene Expression Omnibus (GEO, http://www.ncbi.nlm.nih.gov/geo/), ArrayExpress http://www.ebi.ac.uk/microarray-as/ae/ and Stanford Microarray Database (SMD, http://genome-www5.stanford.edu/, as well as researchers' and institutions' websites. The use of these datasets is not exhausted, when used wisely they may yield a depth of information. Demand has increased to effectively utilise these datasets in current research as additional data for analysis and verification.

Meta-analysis refers to an integrative data analysis method that traditionally is defined as a synthesis or at times review of results from datasets that are independent but related [1]. Meta-analysis has ranging benefits. Power can be added to an analysis, obtained by the increase in sample size of the study. This aids the ability of the analysis to find effects that exist and is termed 'integration-driven discovery' [2]. Meta-analysis can also be important when studies have conflicting conclusions as they may estimate an average effect or highlight an important subtle variation [1,3].

There are a number of issues associated with applying meta-analysis in gene expression studies. These include problems common to traditional meta-analysis such as overcoming different aims, design and populations of interest. There are also concerns specific to gene expression data including challenges with probes and probe sets, differing platforms being compared and laboratory effects. As different microarray platforms contain probes pertaining to different genes, platform comparisons are made difficult when comparing these differing gene lists. Often the intersection of these lists are the only probes to be retained for further analysis. Moreover, when probes are mapped to their 'Entrez IDs' [4] for cross platform comparisons often multiple probes pertain to the same gene. Due to reasons ranging from alternative splicing to probe location these probes may produce different expression results [5]. Ideal methods for aggregating these probe results in a meaningful and powerful way is currently the topic of much discussion. Laboratory effects are important because array hybridisation is a sensitive procedure. Influences that may effect the array hybridisation include different experimental procedures and laboratory protocols [6], sample preparation and ozone level [7]. For more details of the difficulties associated with microarray meta-analysis please refer to Ramasamy et al. 2008 and other works [5,8-12].

We propose a new meta-analysis approach and provide a comprehensive comparison study of available meta-analysis methods. Our method, 'meta differential expression via distance synthesis', (mDEDS) is used to identify differentially expressed (DE) genes which extends the DEDS method [13]. This new method makes use of multiple statistical measure across datasets to obtain a DE list, but becomes a novel tool, with respect to DEDS with the ability to integrate multiple datasets. Hence this meta-method concatenates statistics from datasets in question and is able to establish a gene list. Such integration should be resilient to a range of complexity levels inherent in meta-analysis situations. The strength of mDEDS as a meta-method over DEDS as a method for selecting DE genes is highlighted by comparing these two approaches to one another in a meta-analysis context. Throughout this paper the statistics used within mDEDS and DEDS are the t and modulated t statistic [14], SAM [15], the B statistic [16] and fold-change (FC) statistic, although any statistic can be chosen.

We also perform a comparison study of meta-analysis methods including the Fisher's inverse chi-square method [17], GeneMeta [2,18], Probability of Expression (POE) [19], POE with Integrative Correlation (*IC*) [20], RankProd [21] (the latter four are available from Bioconductor) and mDEDS as well as two naive methods, 'dataset cross-validation' and a 'simple' meta-method. For meta-methods with several varying parameters, we have made use of the suggested or default options.

The performance of the different meta-analysis methods is assessed in two ways, through a simulation study and through two case studies. For the simulation study performance is measured through receiver operating characteristic (ROC) curves as well as the area under these ROC curves (AUC). The two different case studies vary in complexity and performance are assessed through predication accuracy in a classification framework. Warnat et al. [22] uses validation to evaluate performance while using multiple datasets. Our validation method differs from their process slightly. Their method takes a random selection of samples from multiple datasets to obtain a test and training set. We retain to original datasets, leaving them complete. Our method aims to simulate real situations where an additional dataset would need to be classified after a discriminate rule was developed. Although within this paper mDEDS is used in a binary setting, mDEDS is a capable multi-class meta-analysis tool, which is a concept examined by Lu et al. [23].

It is possible to consider meta-analysis at two levels, 'relative' and 'absolute' meta-analysis. 'Relative' meta-analysis looks at how genes or features correlate to a phenotype within a dataset [10]. Multiple datasets are either aggregated or compared to obtain features which are commonly considered important. Meta-methods pertaining to this method include Fisher's inverse chi-square, GeneMeta, RankProd and the 'dataset cross-validation' meta. 'Absolute' meta-analysis seeks to combine the raw or transformed data from multiple experiments. By increasing the number of samples used, the statistical power of a test is increased. Traditional microarray analysis tools are then used on these larger datasets. The 'simple' meta method is an example of 'absolute' meta-analysis approach.

In this paper we will begin by describing existing meta-analysis methods, then we will outline our proposed mDEDS method. This is followed by the comparison study, where publicly available datasets are combined by different meta-analysis methods, examining their ability under varying degrees of complexity, as well as comparing mDEDS to DEDS. Finally, we provide discussion and conclusions of results.

### Existing meta-analysis methods

Let *X *represent an expression matrix, with *i *= 1, *..*., *I *genes and *j *= 1, *..*., *N *samples. If there are *k *= 1,..., *K *datasets, *n_k _*represents the number of samples in the *k*th dataset. For simplicity, and without loss of generality, we focus on dichotomous response; i.e., two-group comparisons. We designate groups as treatment *T *and control *C*. For two-channel competitive hybridization experiments, we assume that the comparisons of log-ratios are all indirect; that is we have *n_T _*arrays in which samples from group *T *are hybridized against a reference sample *R*, and we can obtain *n_T _*log-ratios,  = log_2_(*T_j_/R*); *j *= 1, *..*., *n_T _*from group T. In an identical manner *n_c _*log-ratios are also calculated from group *C*. For Affymetrix oligonucleotide array experiments, we have *n_T _*chips with gene expression measures from group *T *and *n_C _*chips with gene expression measures from group *C*.

#### Fisher's inverse chi-square

Fisher, in the 1930 s developed a meta-analysis method that combines the p-values from independent datasets. One of a plethora of methods for combining the p-values [17], is the Fisher summary statistic,(1)

which tests the null hypothesis that for gene *i*, there is no differences in expression means between the two groups. The p-value *p_ik _*is the p-value for the *i*th gene from the *k*th dataset. In assessing *S_i_*, the theoretical null distribution should be . It is also possible to extend the Fisher methods by producing weights for different datasets based on, for example, quality.

#### GeneMeta

One of the first methods that integrates multiple gene expression datasets was propose by Choi et al. [2] who describe a t-statistics based approach for combining datasets with two groups. An implementation of this method is found in GeneMeta[18] an R package containing meta-analysis tools for microarray experiments.

Choi et al. [2] described a meta-analysis method to combine estimated *effect-sizes *from the *K *datasets. In a two group comparisons, a natural effect size is the *t*-statistics. For a typical gene *i*, the effect size for the *k*th dataset is defined as(2)

where  and  represent the means of the treatment and the control group respectively in the *k*th study. *S_pk _*is the pooled standard deviation for the *k*th dataset.

For *K *number of observed effect sizes, Choi et al. [2] proposed a random effects model

where *μ *is the parameter of interest, *s_k _*denotes the within study variances and *δ *~ *N*(0, *τ*^2^) represents the between study random effects with variance *τ*^2^. Choi et al. [2] further mentioned that when *τ*^2 ^= 0, *δ_k _*denotes the between study effect in a fixed effect model. The random effects model is then estimated using a method proposed by DerSimonian and Laird [24] and a permutation test is used to assess the false discovery rate (FDR).

#### metaArray

The R package metaArray contains a number of meta-analysis methods. The main function is a two steps procedure which transformed the data into a probability of expression (POE) matrix [19] and followed by a gene selection method based on 'integrative correlation' (*IC*) [20].

Given a study, the POE method transforms the expression matrix *X *to a matrix *E *that represents the probability of differential expression. Each element in the matrix *E_ij _*is defined as the chance of multiple conditions present across *N *samples within gene *i*. The transformed matrix, *E*, consists of three values -1, 0, 1 that represent the conditions 'under-expressed', 'not differentially expressed' and 'over-expressed'. After the transformation into a POE matrix, genes of interest are established using *IC *[20]. Notice that this integrative correlation method is not restricted to be used with a POE matrix. The method IC begins by calculating all possible pairwise Pearson correlations (, where *i *≠ *i*') between genes (*i *and *i*') across all samples within a dataset *k*. Thus, we generated a pairwise correlation matrix *P *with  rows representing the number of pairwise correlation and *K *columns representing the number of datasets.

For a selected pair of datasets *k *and *k*', let us denote  and  as means of the correlations per study. Gene-specific reproducibility for gene *i *is obtained by only considering comparisons that contain the *i*th gene. That is(3)

where *i *≠ *i*'. When more than two datasets are being compared, all integrative correlations for a particular gene are aggregated. This method provides a combined ranking for genes across *K *datasets.

In this comparison study, two metaArray results are used. Distinction will be made between them using the terms 'POE with *IC*' and 'POE with *Bss/Wss*' to indicate what type of analysis was performed after the construction of the POE matrix.

#### RankProd

RankProd is a non-parametric meta-analysis method developed by Breitling et al. [21]. Fold change (FC) is used as a selection method to compare and rank the genes within each dataset. These ranks are then aggregated to produce an overall score for the genes across datasets, obtaining a ranked gene list.

Within a given dataset *k*, pairwise FC (pFC) is computed for each gene *i *as(4)

producing *n_T _*× *n_C _pFC_l,m _*values per gene with *l *= 1, *..*., *n_T _*and *m *= 1, *..*., *n_C_*. The corresponding pFC ratios are ranked and we may denote this value as *pFC*_(*i;r*)_, where *i *= 1, *..*., *I *represents the number of genes and *r *= 1, *..*., *R *represents the number of pairwise comparisons between samples. Then the rank products for each gene *i *is defined as(5)

Expression values are independently permuted *B *times within each dataset relative to the genes, the above steps are repeated to produce  where *b *= 1, ..., *B*. A reference distribution is obtained from all the  values, and the adjusted p-value for each of the *I *genes is obtained. Gene considered significant are used in future analysis.

#### Naive meta-methods

Two forms of naive meta-methods are used in the comparison study. The 'simple' meta-method takes the microarray expression matrices and simply combines the datasets together, forming a final matrix made up of all samples with no expression adjustment. The 'dataset cross-validation' meta-method takes one datasets and applies the analysis, these results are then used by the other dataset(s) with the expectation that the results will be transferable. In a classification context this means that one dataset is used for feature selection and development of the discriminant rule and we predict the outcome of the other dataset(s) via this rule.

## Method

### Algorithm - mDEDS

'Meta differential expression via distance synthesis' (mDEDS) is a meta-analysis method that makes use of multiple statistical measures to obtain a DE list. It is aided by 'Differential Expression via Distance Synthesis', DEDS [13] which is designed to obtain DE gene lists. Example DE measures include standard and modulated-t stat [14], fold change, SAM [15] and the B-statistic, amongst many others. The concept behind the proposed meta-method considers that truly DE genes should be selected regardless of the platform or statistic used to obtain a DE list.

The true DE genes should score highly within a set of non-dominated genes, both within a dataset using DE measures and also between datasets when the same DE measures are used on different datasets across different platforms. Consistently high ranked genes are then considered DE via mDEDS. This method endeavours to be robust against both measure specific bias, when different measure produce significantly different ranked lists, and platform specific bias where particular platforms produce results that are more favourable to particular gene sets.

1. Let there be *k *= 1, ..., *K *datasets and *g *= 1, ..., *G *appropriate (DE measuring) statistics, hence there will be *K *× *G *statistics for each of the *i *= 1, ..., *N *genes. Let *t_ikg _*be the statistic for the *i*th gene, from the *k*th dataset for the *g*th DE measure. Assuming large values indicate increased DE genes, let the observed coordinate-wise extreme point be(6)

2. Locate the overall (observed, permutation) extreme point E:

(a) Each of the *K *datasets is permuted B times by randomly assigning *n_T _*arrays to class 'T' and *n_C _*arrays to class 'C', producing b = 1,... B sets of *K *datasets. For each permuted datasets the *G *number of DE statistics are recalculated yielding . Obtain the corresponding coordinate-wise maximum:(7)

(b) Obtain the coordinate-wise permutation extreme point *E_p _*by maximizing over the *B *permutations,(8)

(c) Obtain *E *as the overall maximum: *E *= max(*E_p_, E*_0_).

3. Calculate a distance *d *from each gene to *E*. For example, one choice for a scaled distance is(9)

where MAD is the median absolute deviation from the median. Order the distances, *d*_(1) _≤ *d*_(2) _≤ ... ≤ *d*_(*N*)_.

Batch correction can be performed by mDEDS, by substituting datasets with 'batch groups' (see Discussion).

### Comparison study

Eight meta-analysis methods are compared using a simulated dataset and two cases studies comprising of six publicly available datasets, pertaining to three breast cancer and three lymphoma datasets. The purpose of the comparison study is to establish how these meta-analysis methods perform under varying degrees of dataset complexity. Dataset complexity refers to the level of difficulty present when combining multiple datasets. For example datasets being produced on the similar platforms (for example different Affymetrix platforms) are less complex to analyse via meta-analysis then when analysing results across very different platforms. For this comparison paper two levels of dataset complexity are considered. *Case study 1*, implemented by the breast cancer data contains datasets from identical Affymetrix chips, this is considered 'similar platform meta-analysis'. *Case study 2 *which makes use of the lymphoma datasets contains samples that are hybridised using long oligo two colour platforms, Affymetrix chips and the 'Lymphochip' [25], this is considered 'disparate platform meta-analysis'.

For the publicly available data, probe sets for each platform are mapped to the relative 'Entrez IDs' [4]. Where multiple probes pertained to the same gene the mean gene expression level is used. Probes with unknown 'Entrez IDs' are discarded. Only the intersection of platform gene lists are used in further analysis. Data is imputed using KNN imputation with k = 10. Data analysis performed using R.

#### Performance assessment

Assessing the performance of different meta-analysis methods is important to evaluate and compare methods. Although important, performance assessment of meta-analysis methods is non-trivial. Typically meta-analysis methods will be evaluated using pre-published gene lists and noticing the concordance of the obtained DE gene list and published material, this process however is subject to publication bias. To avoid such biases two forms of performance assessment will be applied in this paper.

1. Receiver operating characteristic curves (ROC): For the simulated data where the 'true' DE gene list is known, meta-analysis performance is measured via ROC curves. ROC curves are created by plotting the true positive rates verses the false positive rates for the obtained DE genes. Performance is indicated by how close the plots are to the upper left hand corner of the ROC space. The AUC is also used as a comparison tool, with AUC values close to one indicating an accurate DE list. Because of the design of the simulation study the 'cross-validation' meta-analysis method can not be used.

2. Prediction accuracy: For the case studies, prediction accuracy under a classification framework is used to asses performance of the DE list. We will use the term DE list for the consistency of this manuscript, although strictly speaking in a classification framework such gene lists are known as feature gene lists. To classify within the case studies, each consisting of three independent datasets, two datasets are combined via the meta-analysis methods and DE genes are selected. When DE gene selection is not part of the meta-analysis approach DE genes are ranked via 'between sum of squares over within sum of squares' *Bss*/*Wss *[26]. Using these two datasets, a discriminant rule is constructed by diagonal linear discriminant analysis (DLDA) [26]. The third independent dataset is classified using this rule. The ability for the meta-analysis method to collaborate information from the two distinct datasets is reffected in the ability to classify the third. Prediction accuracy is used because the 'true' DE list is not known. In these case studies, performance can only be judged relative to the other compared methods.

#### Simulation study

To evaluate the performance of the different meta-analysis methods, data was simulated to represent three separate gene expression datasets. The simulation approach is adapted from an approach presented by Ritchie et al. [27]. A non-parametric bootstrap simulation is used where a matrix of non-differentially expressed gene expression data is sampled from three different datasets. This 'background' noise contains the latent characteristics of an actual microarray data yet contains no biologically DE genes. Samples are constructed with replacement from the original data, such that an even binary class distribution is established.

DE genes are simulated via a 2 fold increase in fold change. Two types of DE genes are simulated, 'true' DE genes, and 'platform specific' DE genes. 'True' DE genes are identical genes within each dataset, representing biologically relevant DE genes. 'Platform specific' DE genes simulate platform bias apparent within DE genes from microarray experiments [28] and are randomly selected from the genes in the datasets, with the exclusion of the 'true' DE genes. This simulation taps into the important notion that a powerful meta-analysis tool will have the ability to correctly distinguish a true DE gene which is DE across multiple platforms from a DE gene which is simply a platform phenomena.

#### Case study 1 - Breast cancer: Similar platform meta-analysis

Three publicly available Affymetrix datasets are used for the breast cancer study, all three datasets use the affymetrix platform U133A. Classification of the breast cancer samples aims to distinguish between the sample's estrogen receptor (ER) status (+ve or -ve) as determined by the sample information provided with the datasets, we refer readers to the original manuscripts for more details regarding this status. In this case ER status is being used simply as a response variable common throughout all considered datasets, it should be understood that predicting ER status using gene expression data is not the same as immunohistochemistry. These datasets include the Farmer et al. dataset [29] (GSE1561) which utilises the Affymetrix U133A platform with 49 samples, comprising of 27 +ve and 22 -ve samples. The Loi et al. dataset [30] contains Affymetrix samples from three platforms, U133 (A,B) and U133plus some of which underwent treatment and some which did not. Samples from platform U133A which did not experience any treatment are used in this study, which totalled 126 with 86 +ve and 40 -ve samples (GSE6532). Ivshina et al. [31], developed breast cancer samples on Affymentrix U133A arrays, 200 in total corresponding to 49 +ve and 151 -ve samples (GSE4922). The performance of the meta-analysis methods employed in a 'similar platform meta-analysis' context was assessed via classification. The Farmer et al. and Ivshina et al. datasets were combined via meta-analysis and used to obtain a DE gene list and construct a classification model. The Loi et al. dataset was classified using this gene list and discriminant rule.

#### Case study 2 - Lymphoma: Disparate platform meta-analysis

An original lymphoma dataset was obtained from the Department of Haematology and Stem Cell Transplant at St Vincent's Hospital Sydney (which will be referred to as SVH). Gene expression levels have been gathered from 60 patients presenting with lymphoma cancers, 37 of these samples are Follicular Lymphoma (FL) and 23 samples are Diffuse Large B-Cell Lymphoma (DLBCL). Human 19000 oligo array slides from the Adelaide microarray consortium were used to obtain microarray expressions. Two well known publicly available datasets were also analysed. The Shipp et al. data [32] contains 19 FL and 58 DLBCL samples, hybridised using the Affymetrix platform HU6800. Alizadeh et al. [25] also contains 19 FL samples and 27 DLBCL samples, hybridized to the 'Lymphochip' which is a custom designed cDNA microarray. The performance of the meta-analysis methods employed in a 'disparate platform meta-analysis' context was also assessed via classification. The Shipp et al. and Alizadeh et al. datasets were combined via meta-analysis and used to obtain a DE gene list as well as construct a classification model. The SVH dataset was classified using this gene list and classification rule.

#### Case study 3 - mDEDS versus DEDS

To establish the success of mDEDS as a meta-analysis method beyond the capabilities of DEDS, DEDS and mDEDS are compared. The strength of DEDS comes from its ability to synthesise results from a range of statistics, mDEDS goes beyond this to consider results from a range of statistics across multiple datasets. DEDS is a method for selecting DE genes and to this end was used in the simple meta-method described in the 'Existing meta-analysis methods' section. Datasets from both the breast cancer study and the lymphoma study were used in the comparison of these meta-methods with the Loi et al. and the SVH datasets used as the independent test sets.

## Results

### Simulation

**Figure 1:**
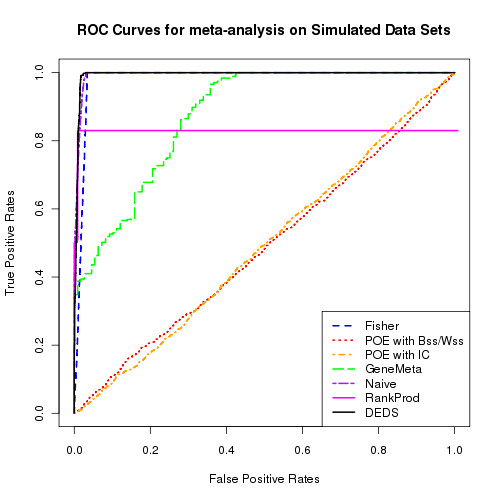
**ROC curves for simulation**. ROC curves for differing meta-analysis methods. GeneMeta, RankProd, POE with *Bss/Wss *and POE with *IC *appear to struggle with obtaining an accurate 'true' DE list, Fisher and mDEDS perform competitively.

Three datasets were simulated, with 150, 100 and 80 samples, each with 20000 genes. The percentage of DE genes varied between 2.5%, 4% and 10%, with half the DE genes on each platform being 'true' and the other being 'platform specific' DE genes. Figure 1 shows the ROC curves for 5% true and 5% platform specific DE genes. These results are indicative of all considered DE percentages. Table 1 contains the AUC values for the three different DE gene percentage levels for the different meta-analysis methods. GeneMeta, RankProd, POE with *Bss/Wss *and POE with *IC *appear to struggle with obtaining an accurate 'true' DE list. Fisher and mDEDS perform competitively with the difference between Fisher, simple and mDEDS reducing as the number of genes in the gene list increases.

**Table 1:** AUC values for simulated and dataset analysis

	AUC		
Meta-method	2.5%	4%	10%
Fisher	0.996	0.993	0.982
POE with *Bss/Wss *	0.489	0.490	0.487
POE with *IC *	0.483	0.492	0.491
GeneMeta	0.861	0.866	0.876
RankProd	0.999	0.998	0.834
Simple	0.998	0.998	0.994
mDEDS	0.998	0.998	0.994

The AUC values for the simulated datasets, for each meta-analysis method. DE genes are simulated at 2.5%, 4% and 10% levels, with half the genes being 'true' DE genes and the other half being 'platform specific' DE genes

### Case study 1 - Breast cancer: Similar platform meta-analysis

**Figure 2:**
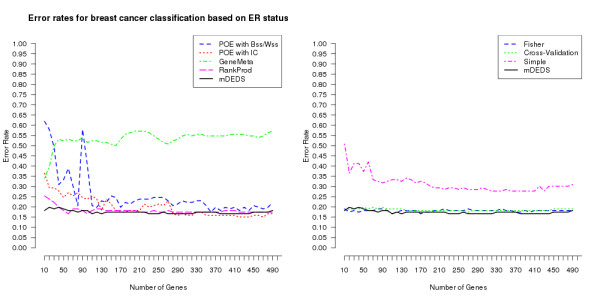
**Breast cancer classification**. Plots of error rates in the binary classification of three breast cancer datasets as the number of genes used to build the classifier varies from 10 to 500. Classification error rates are displayed for the 8 different meta-analysis approaches. Plots are split into two sub-plots for reading ease, mDEDS appears in both for comparative purposes.

Figure 2 displays the error rates for the classification of the Loi et al. dataset, the number of DE genes used to build the classification model varies across the horizontal axis. The mean error rates can be found in Table 2. The majority of the applied meta-methods successfully capture the DE genes across all three Affymetrix platforms to distinguish between the binary classification of positive and negative ER status, with the notable exceptions of GeneMeta and the simple meta-method. Both POE methods become more reliable meta-methods as the number of genes used to build the classifier increases. RankProd, Fisher, the cross-validation meta-method and mDEDS produce consistently relatively low classification errors for this similar platform analysis. When Farmer et al. and Ivshina et al. were used as the independent test sets, results from the meta-analysis methods were similar (results not shown).

**Table 2:** Breast cancer classification error rates

Meta-Method	Mean Error
Fisher	0.182
POE with *Bss/Wss *	0.257
POE with *IC *	0.199
GeneMeta	0.534
RankProd	0.182
Simple	0.314
Cross-Validation	0.186
mDEDS	0.174

Mean of error rates in the binary classification of three breast cancer datasets using DLDA

### Case study 2 - Lymphoma: Disparate platform meta-analysis

**Figure 3:**
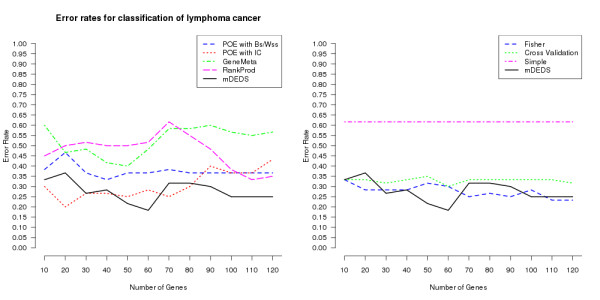
**Lymphoma cancer classification**. Plots of error rates in the binary classification of three lymphoma cancer datasets as number of feature used in classification varies from 10 to 500. Classification error rates are displayed for the 8 different meta-analysis approaches. Plots are split into two sub-plots for reading ease, mDEDS appears in both for comparative purposes.

Figure 3 shows the error rates for the prediction of the SVH dataset. This study examines the different meta-methods across highly varying platforms (both cDNA and Affymetrix). The Fisher's inverse chi-square and POE with *IC *meta-methods perform well under such conditions, conversely GeneMeta, POE with *Bss/Wss *and RankProd appear to struggle in DE gene selection. However, mDEDS can still utilise these different experiments purposefully, producing the lowest mean error rate (Table 3), and a very competitive classifier. When Shipp et al. and Alizadeh et al. were used as the independent test sets, results from the meta-analysis methods were similar (results not shown).

**Table 3:** Lymphoma cancer classification error rates

Meta-Method	Mean Error
Fisher	0.276
POE with *Bss/Wss *	0.375
POE with *IC *	0.301
GeneMeta	0.525
RankProd	0.475
Simple	0.617
Cross-Validation	0.329
mDEDS	0.277

Mean of error rates in the binary classification of three Lymphoma datasets using DLDA.

### Case study 3 - mDEDS versus DEDS

Table 4 shows the mean error rates of the breast cancer and the lymphoma datasets when classified using mDEDS and the simple meta-method with DEDS as the method for selecting DE genes. It is apparent from this table that DEDS is not capturing the DE genes across the multiple datasets to distinguish the two classes being compared, although mDEDS is acting as a successful meta-method.

**Table 4:** mDEDS versus DEDS

Meta-Method	Breast cancer study	Lymphoma study
	Mean error	Mean error
Simple meta with DEDS	0.441	0.617
mDEDS	0.174	0.277

Mean of error rates when comparing mDEDS to the simple meta-methods when DEDS is used as a feature selection method. Performance is assessed in the binary classification of the breast cancer and lymphoma datasets using DLDA.

## Discussion

The simulation study coupled with the two cases studies of varying meta-analysis complexity offers insight into the eight meta-analysis methods compared in this paper. It is important to validate meta-analysis methods, although at times this is difficult to perform. Some meta-methods are simple variants of common classical statistical methods, others offer more sophisticated responses to specific issues faced in the microarray environment. A large proportion of meta-research deals with DE genes and the process of obtaining a DE list from multiple datasets. Unfortunately DE gene lists are illusive because the biological DE gene lists are not known. Often for validation purposes DE lists are compared to other published DE lists with the level of congruency indicative of the success of the meta-method. This method suffers from publication bias [26] as one is continuously publishing pre-published information, with little validation to the variations that are occuring. An alternative assessment criteria utilizing the classification framework offers an intuitive validation process with interpretable results. Classification performance relies heavily on the accuracy of the classifier's feature list, which is traditionally taken from the DE list. Within this meta-analysis study independent dataset validation classification was performed, using DLDA. DLDA was chosen as Dudoit et al. [33] found that DLDA was an effective, efficient and accurate classifier for microarray data. This study could have been conducted using any number of classifier s provided feature selection is not performed implicitly by the classifier. The varying DE list obtained from the meta-methods are the only varying component in the comparison. Therefore a reduction in classification error can be attributed to the meta-method.

Meta-analysis offers a way to enhance the robustness of microarray technology. The 'dataset cross-validation' meta-analysis approach observed within this study encapsulates a very real problem with microarrays; gene lists selected from one platform or study have a limited ability to be transfered. This is highlighted by their inability to be used to classify samples generated by another platform or study, as demonstrated by the 61.7% error rate obtained via this method (Table 3). For both the breast cancer and lymphoma case studies some meta-analysis approaches were able to increase the accuracy of cross platform classification, at times the error reduced by as much as 33% which can be seen in Table 3. This indicates that the added power through meta-analysis produces more robust and reliable results, eventuating in a gene list that is not platform dependent but truly indicative of the disease.

Cross platform meta-analysis multiplies the level of complexity in this particular analysis paradigm. The meta-analysis complexity is suggestive of the meta-method one should employ. Within this study we have used two levels of meta-analysis complexity, (i) when meta-analysis is performed across similar platforms, for example Affymetrix with Affymetrix, (ii) when meta-analysis is performed across disparate platforms, for example Affymetrix with oligo arrays.

The breast cancer case study uses datasets from three identical Affymentrix platforms. Affymetrix's development and processing protocols offer a reduced variability in array comparison [34]. This feature of Affymetrix arrays is highlighted with the success of the cross-validation meta-analysis method, producing a relatively low mean error rate within the breast cancer study. In this case POE with both *Bss/Wss *and *IC*, Fisher's inverse chi-square and RankProd were able to classify competitively, hence they are able to highlight between dataset DE genes. RankProd's success in this circumstance is similar to the findings by Hong et al. [3] where RankProd is shown to be powerful in both simulated and Affymetrix based meta-analysis studies.

The lymphoma case study aims to distinguish between FL and DLBCL subtypes and the datasets used makes this analysis more complex. Both cDNA and oligonucleotide arrays are compared. These platforms vary remarkably with differences ranging from probe length to the presence of reference samples. As the complexity of the meta-analysis rises POE with *Bss/Wss*, GeneMeta and RankProd struggle to obtain a gene list robust enough for cross platform classification. Two different reasons could attribute to the depletion in accuracy of the meta-methods as the level of complexity increases. The meta methods could be over-fitting the data, methods that model the data are particularly susceptible to this, for example GeneMeta. Conversely, some feature selection methods may not capture the complexity of the data, this is potentially occuring in the POE with *Bss/Wss *case. Fisher's inverse chi-square meta approach does not take into consideration the actual intensities of each spot on the microarray, albeit at times this method is ideal, for example when individual intensities are unknown, or when the characteristics of the study vary greatly [35]. This particular characteristic of Fisher's inverse chi-Square method is highlighted by the more complex lymphoma case study producing lower relative classification errors than when used in similar platform breast cancer analysis.

Within both complexity environments mDEDS is able to perform DE analysis well, as this method makes use of the different datasets but does not try to fit a full parametric model to the data. Our proposed mDEDS uses multiple statistical measures while developing its ordered gene list. Using multiple measures aids robustness as more of the variability can be encapsulated within the meta-method. The success of mDEDS over DEDS as a meta-method highlights that the method of combining different statistics across datasets aids in the meta-analysis process. It is possible that the multiple platforms and multiple measures draw enough diversity to begin to transcend cross platform variability and produce a reliable gene list. The variation in some of the meta-method's abilities within classification suggests that different tools are beneficial depending on the researcher's current meta-analysis project.

One may speculate that mDEDS can be used in a batch correction context. Batch effect is a term given to non-biological experimental variation that occurs throughout an experiment. In most cases batch effects are inevitable as non-biological variations are observed simply through multiple, apparently identical, amplification and hybridisation. Staggering ones hybridisation process is a practical reality of microarray experiments for two main reasons: (i) data is often prospective and may be collected and processed in stages, (ii) there is a limit to the number of samples that may be amplified and hybridised at one time [36] hence forcing batches to form. As a result, powerful batch correction methods are vital for microarray research. One could consider batches obtained separately with time delays, for example a year, as separate batches, which resemble individual datasets on similar platforms. By using mDEDS one can borrow strength from the multiple batches yet avoid particular batch bias.

There are still many open questions within the meta-analysis paradigm. For example questions pertaining to mismatched probe sets across platforms and the handling of multiple probes for the same genes. More research within these areas would greatly aid meta-analysis for microarrays and ones ability to make use of the current plethora of information laying dormant in these public repositories. However, once more of these type of tools for meta-analysis have been developed, meta-analysis will save time, money and scientific resources.

## Conclusion

We compared eight meta-analysis methods, which comprise of five existing methods, two naive approaches and our novel approach, mDEDS. Integrating datasets within microarray analysis has copious and clear advantages. This study adds in establishing which meta-analysis methods are more successful in their approach by comparing multiple meta-analysis methods, including the Fisher's inverse chi-square, GeneMeta, POE with *Bss/Wss*, POE with *IC*, RankProd, a 'dataset cross-validation' meta and a 'simple' meta.

Our proposed method; mDEDS, has performed competitively and at times better than currently available meta-analysis methods. ROC curves were used as a comparison in a simulated study and prediction accuracy within classification was used as an evaluation tool in two real biological case studies. These case studies differ in complexity regarding data being combined, the first demonstrating the combining of three datasets from similar platforms (different Affymetrix chipsets) and the second combining datasets from Affymetrix, cDNA and the Lymphochip.

In both classification comparisons mDEDS was used as a feature selection method and produced capable classifiers, with all else held constant. These results, coupled with results from the simulated data, are indicative of mDEDS being used as a powerful meta-analysis method for cross laboratory and platform studies.

## Availability and requirements

The R code for mDEDS is an additional feature within the DEDS package available at http://Bioconductor.org.

## Authors' contributions

AC performed the analysis and wrote the manuscript. YHY conceived the study, supervised the analysis and participated in the preparation of the manuscript. Both authors read and approved the final manuscript.
